# An mHealth App to Support Patients With Psoriasis in Relation to Follow-up Consultations: Qualitative Study

**DOI:** 10.2196/28882

**Published:** 2021-06-08

**Authors:** Bettina Trettin, Dorthe Boe Danbjørg, Flemming Andersen, Steven Feldman, Hanne Agerskov

**Affiliations:** 1 Department of Dermatology and Allergy Centre Odense University Hospital Odense Denmark; 2 Faculty of Health Science Institute of Clinical Research University of Southern Denmark Odense Denmark; 3 Centre for Innovative Medical Technology Odense University Hospital Odense Denmark; 4 Department of Dermatology Aalborg University Hospital Aalborg Denmark; 5 Private Hospital Molholm Vejle Denmark; 6 Wake Forest School of Medicine Winston-Salem, NC United States; 7 Department of Nephrology Odense University Hospital Odense Denmark

**Keywords:** psoriasis, teledermatology, qualitative, video consultations, app, participatory design, mHealth, telehealth, patient-physician relationship, dermatology

## Abstract

**Background:**

Teledermatology has the potential to help deliver health care by transforming the relationship between patients and health care professionals (HCPs), shifting the power of consultation so that patients can become more informed, assertive, and involved in their care. Mobile health (mHealth) is a promising and reliable tool for the long-term management of patients with psoriasis on systemic treatment. In an attempt to facilitate a more patient-centered approach in clinical practice, we designed and developed an mHealth solution to support patients with self-management and empowerment.

**Objective:**

The aim of this study is to explore the experiences and perceptions of patients and health care professionals of using an mHealth solution that was developed using a participatory design approach.

**Methods:**

This was an exploratory qualitative study. Data were collected through semistructured interviews with patients and focus group interviews with HCPs.

**Results:**

All participants found it easy to use the mHealth solution, and the patients found it convenient. Patients’ reflexivity was improved because they could prepare ahead of consultations. Video consultations provided patients with a degree of freedom in their everyday lives, with not having to attend in-person visits. Among the HCPs, there were concerns regarding their medical responsibilities, as they could not assess the patients’ skin as they used to. The mHealth solution required new workflows and procedures that were not part of the existing consultation routines.

**Conclusions:**

The mHealth solution can strengthen the relationship between HCPs and patients and facilitate patients to become more active in their care. Alignment and structure in relation to the selection of eligible patient candidates for being offered the mHealth solution could reduce social health inequalities. In addition, video consultations changed HCPs’ work practice, necessitating new types of skills to communicate with patients.

## Introduction

### Background

Patient-centered health care, including patient empowerment and self-management, can enhance the care of dermatology patients. Although patients often prefer a patient-centered approach, it requires them to be more responsible for their treatment; the approach also requires a different relationship with health care professionals (HCPs) [1]. Thus, patient-centered care increases patients’ responsibilities and may require changes in HCPs’ interactions with their patients. For a chronic complex inflammatory disease such as psoriasis to be managed with a patient-centered approach, patients need to be informed and able to make decisions about treatment options, lifestyle behavior, and comorbidities [2]. However, patients with psoriasis may not have the necessary knowledge [3], and consultations may not fully address patients’ needs [4,5].

Teledermatology (TD) has the potential to help deliver health care by transforming the relationship between patients and health care workers, shifting the power of consultation so that patients can become more informed, assertive, and involved in their care [6]. In an attempt to facilitate a more patient-centered approach in clinical practice, we designed and developed a TD solution to support patients with self-management and empowerment in relationship with HCPs [7]. TD may help optimize psoriasis treatment [8] and is well accepted by both patients and health care workers [9]. TD in the form of a mobile health (mHealth) solution could also be a promising and reliable tool for the long-term management of patients with psoriasis on systemic treatment (eg, biologics), where the course of the disease can be properly monitored and side effects of medications can be detected earlier [10].

### Objectives

This study is part of a participatory design (PD) study exploring how the care and management of patients with psoriasis receiving biological treatment can be promoted by a TD solution. In the first phase of the study, patients’ and HCPs’ needs were identified [4,11]. In the second phase, a TD solution was designed in close collaboration with the patients, HCPs, information technology designers, and the research team [7]. The TD solution is an mHealth app designed to support needs, both during in-person consultations and by offering live interactive consultations, thereby reducing patients’ in-person visits. This study reports on phase 3, in which the app was evaluated and tested in clinical practice and in patients’ daily lives. Thus, the aim of this study is to explore the experiences and perceptions of patients and HCPs of using an mHealth app that was developed using a PD approach.

## Methods

### The Process of Developing the TD Solution

As PD was the theoretical basis of the study, the TD solution was designed with the participation of patients, HCPs, information technology designers, and researchers. The design of the solution was guided by the needs of its users and the result was the design of an app. The app included a knowledge database with information, videos, and a podcast relevant to patients. The app included 2 questionnaires for patients to complete before consultations: the Dermatology Quality of Life Index (DLQI) and a second questionnaire, which was named “preparation before consultation.” It included patient-reported outcome questions and a free-text space for questions or comments to capture topics that patients wanted to discuss at consultations. Furthermore, the TD solution provided patients with the option of a video consultation. The overall aim of the TD solution was to facilitate a more patient-centered approach that would give patients the opportunity to contribute to the consultation agenda. The app was designed to support patients during their daily lives with psoriasis, when attending in person, and when offered a video consultation. Before testing the app in clinical practice, the HCPs received information about the app and its content, how to access the questionnaires and patient data, and training on how to interact during video consultations.

### Design

This study was a qualitative explorative study that used a phenomenological-hermeneutic approach [12]. Semistructured telephone interviews with patients and focus group interviews with HCPs were conducted to gain insight into their experiences and perspectives of using the mHealth app in their daily lives and in clinical practice, respectively [13].

### Setting

The study was conducted at an outpatient clinic at a university hospital in Denmark, where the majority of patients with psoriasis receiving biological treatment have regular follow-ups every 3 months with both a nurse and a medical doctor. However, a minority of patients were offered telephone consultations or a nursing consultation twice a year. Patients were monitored by laboratory tests; however, these were required only when patients were scheduled for an in-person visit. Laboratory tests do not require an appointment at the hospital, as the patients can choose to have them taken by their general practitioner or a local hospital close to where they live. The results of the laboratory tests were then available in the patients’ personal electronic medical records at the hospital. Furthermore, patients do not have to travel to the hospital to pick up their medication; instead, they can receive it at their hospital. Patients included in the study tested the app, its content, and features for 3 months. The patients were invited by HCPs to test the solution during a scheduled in-person visit at the outpatient clinic, where the app was installed on their own devices and where they received information about the content and features by the first author and a medical secretary.

### Participants

#### Patients

Patients were recruited in March 2020 by HCPs during in-person follow-up consultations. Inclusion criteria were as follows: Danish-speaking patients, aged >18 years, with psoriasis and patients receiving biological treatment. Of the 17 patients invited to participate, 1 patient declined because he found the TD solution to be too complicated. One patient dropped out because he found it too burdensome to answer the questionnaires. A total of 15 patients were included. The test period was 3 months and lasted until the next scheduled consultation. In total, 10 patients were scheduled for a video consultation, and 5 patients were scheduled for an in-person visit (Table 1). Moreover, 14 patients participated in the interviews, as 1 patient did not respond despite several attempts.

**Table 1:** Participants’ characteristics (N=14).

Variables			Values
Age (years), median (range)			45 (27-67)
**Sex, n (%)**			
	Male	12 (76)	
	Female	2 (14)	
**Employment status, n (%)**			
	Employed	11 (79)	
	Retired	2 (14)	
	Not working	1 (7)	
**Relationship, n (%)**			
	Single	2 (14)	
	In a relationship	12 (76)	
	Widowed	0 (0)	
**Treatment, n (%)**			
	Ustekinumab	9 (65)	
	Adalimumab	3 (21)	
	Secukinumab	2 (14)	
Treatment years, median (range)			5.2 (0.5-14)
DLQI^a^, median			1.8
PASI^b^, median			0.7
Previously received a telephone consultation, n (%)			2 (14)
Received a video consultation, n (%)			9 (65)
Received an in-person visit, n (%)			5 (35)

^a^DLQI: Dermatology Quality of Life Index.

^b^PASI: Psoriasis Area Severity Index.

#### Health Care Professionals

Both nurses and medical doctors who were presumed to have used the TD solution at consultations were invited to participate in the focus group interviews. Thus, the recruitment was based on experience with the use of the mHealth solution and not on maximum variation among the participants, as this was not possible given the limited number of eligible participants. Of the 15 HCPs invited, 6 did not participate. Reasons for not participating were as follows: 2 were absent due to sickness, 1 was not able to attend due to other work, 1 had not used the app, and 2 were not present on the day the focus groups were scheduled. In total, 9 participants participated and were divided into 2 groups (Table 2). As some of the consultations in the test were performed by nurses only and some of the consultations were performed by both nurses and physicians, the focus groups were divided to match this. Therefore, 1 focus group consisted of both nurses and physicians, and 1 focus group consisted of only nurses.

**Table 2:** Characteristics of the health care professionals included in the focus groups (n=9).

Variables				Values
**Focus group 1**				
	**Sex, n (%)**			
		Male	2 (50)	
		Female	2 (50)	
	**Occupation, n (%)**			
		Medical doctor	2 (50)	
		Nurse	2 (50)	
	Experience of treating patients with psoriasis (years), median (range)			5.6 (3-7)
**Focus group 2**				
	**Sex, n (%)**			
		Male	0 (0)	
		Female	5 (100)	
	**Occupation, n (%)**			
		Medical doctor	0 (0)	
		Nurse	5 (100)	
	Experience of treating patients with psoriasis (years), medium (range)			8.2 (4-13)

### Interviews

In total, 14 semistructured interviews were conducted with patients who had completed the test phase to explore their experiences and perceptions of the app and, thereby, to gain insight into their experiences of having used the solution [13]. Due to the COVID-19 pandemic and the related restrictions, the interviews were conducted by telephone. The first author recruited all the patients face-to-face before the lockdown of the society, and thus, all participants had met the researcher conducting the interviews [14]. When scheduling the interviews, patients were advised to be placed in a nondisturbed room. An interview guide was developed to explore the patients’ experiences, impression, and acceptance of the app and its impact on their everyday lives. The interview guide was developed to ensure that participants could share their experiences and perspectives on the app features, function, layout, and comprehensibility, and suggestions for further development. Examples of questions asked included the following: “Please, tell me how you experienced using the app?” and “Can you describe in what way the app influenced your consultation?”

### Focus Groups

The focus groups were conducted during work hours at the outpatient clinic. A semistructured interview guide was used to facilitate reflections on the use of the mHealth solution. Examples of questions asked to facilitate these reflections included the following: “How do you experience the opportunity to provide care and treatment during video consultations?” and “How do you experience the opportunity to provide care and treatment when you include the patient’s app responses at in-person consultations?” Participants were asked to discuss these questions with each other, rather than addressing or answering the moderator [15]. This interaction between participants in a focus group is essential because it creates room for the interviewees to reflect, by exploring each other’s perspectives [15]. The first author was the moderator, and the last author acted as an external observer; took notes on nonverbal communication; and asked questions to clarify issues raised, when necessary.

### Ethical Considerations

All participants were informed about the study and received both oral and written information in accordance with the Declaration of Helsinki [16]. The patients provided written consent to participate and to be contacted after the interview. The HCPs provided written consent to participate in the focus group interviews. The study was approved by the Danish Data Protection Agency (2012-58-0018).

### Data Analysis

All data were gathered into one coherent text, and the analysis was inspired by the theory of narrative and interpretation by Ricoeur [12]. The analysis followed a 3-step process: naïve reading, structural analysis and critical interpretation, and discussion. In the naïve reading step, the text was read several times to establish an initial impression of what the text was about. In the structural analysis and critical interpretation step, units of meaning (what is said) and units of significance (what the text speaks about) were identified [17]. At this stage, the transcripts were viewed objectively by abstracting the units of meaning from the text as a whole to create distanciation from the text [17,18]. In a dialectical process between explanation and understanding, 3 main themes emerged. Findings from the structural analysis step were subsequently interpreted and discussed in relation to the theory and other research results. The aim of this critical interpretation was to gain an even deeper understanding of the themes that had emerged. The findings were discussed by the entire research team. Reporting was guided by the Consolidated Criteria for Reporting Qualitative Studies [19].

## Results

### Overview

The naïve reading step revealed that the video consultations allowed patients a higher degree of flexibility in the everyday lives of patients, compared with in-person consultations. The patients seemed to appreciate the opportunity to prepare before attending a consultation. However, for both patients and HCPs, using an mHealth app was experienced as a new approach that required training. The structural analysis revealed 3 main themes: (1) preparation increases reflexivity, (2) video consultations increase the much-appreciated attentiveness, and (3) a new approach requires new competencies. FG1 and FG2 refer to focus group 1 and focus group 2, respectively; IIP refers to interviews with patients who had in-person visits; IV refers to interviews with patients who had video consultations; and “P” refers to participant number. The results presented focus on the specific TD solution that was tested and not on the general perception of other TD solutions.

### Preparation Increases Reflexivity

Patients considered it to be able to prepare ahead of either video or in-person consultations as an advantage. The home setting gave them a chance to think about what was important for them to discuss:

When you arrive 10 minutes before you have to be there, you probably don’t think so deeply about those things. Here you have the chance to just sit at home and think, well, maybe I could just hear a little about it or ask about.

IIP, P1

It’s good to be able to set an agenda in advance, if there is something you would like to discuss. And then you are also sure to remember it.

IV, P3

The opportunity to contribute to the consultation agenda through the app led to reflexivity, which was not experienced when not using the app. Before patients had the app, patients had to fill out the DLQI in the waiting room, hand in a urine sample, register at the outpatient clinic, and register their transportation, all of which the patients found to be stressful. The app also assisted patients in remembering what they wanted to discuss. The patients were aware that, in advance of the consultation, the HCPs would have the patients’ agenda and notes. This seemed to heighten the HCPs’ focus on their duty to collaborate and engage with the patients.

The HCPs considered that the questionnaires encouraged the discussion of topics important to patients and clarified what patients wanted to address:

If the patient has doubts or something they would like to discuss, then these are actually good (questionnaires), so that is really good. It sets up a kind of agenda for the consultation that may also be important to them, in addition to that regarding the skin.

Doctor, FG1

Questionnaires used as tools for preparation created a structure for consultations that suited both parties. Despite positive acknowledgment of the questionnaires among the HCPs, it was recognized that it would take time to get used to using questionnaires in all consultations. Thus, it was beneficial to both parties that patients had the chance to reflect ahead of consultations.

### Video Consultations Increase the Much-Appreciated Attentiveness

Both patients and HCPs were skeptical of video consultations and were surprised that they were easier than expected. Patients experienced attentiveness on the part of the HCPs and a sense of having a personal relationship with them:

That was the sense of security of it. It works. And those who sit at the other end you can feel they know what they are dealing with/what they are doing. Yes, I actually felt like I was sitting across from them.

IV, P2

Patients felt safe and confident during video consultations and were not distanced, as expected. However, the situation could be awkward:

I think you have a harder time getting started sometimes, so you sit and wait for the doctor, and then you just sit there and get filmed, like. But then we got talking about what we were supposed to talk about, and got the job done.

IV, P6

Some patients felt unaccustomed to video consultations. However, even those who tended to be averse preferred them over telephone consultations. They felt that visual contact avoided misunderstandings and created feelings of security. In addition, patients considered having a personal contact at in-person follow-up consultations before the video consultations as an advantage.

Attentiveness during video consultations was perceived as both unaccustomed and appreciated among HCPs. The HCPs became acutely aware during video consultations that they needed to demonstrate a different level of attentiveness, as compared with in-person meetings:

Well, the video consultation simply sets the stage so that now you have to look at the patient. And talk to the patient. Because they are there, right there on the table, you know? You cannot just sit there and sort through things. It requires a different preparation for us, and I think that is actually really good.

Nurse, FG2

HCPs were not used to having visual contact, as before video consultations became available, telephone consultations were the only option. Using a telephone gave them the opportunity to arrange other matters during these consultations, such as documenting or reviewing electronic patient records. Some HCPs appreciated being *forced* to be attentive, whereas others perceived it to be more time consuming and challenging. Thus, they felt unused to this but appreciated it.

Among the HCPs, there were some concerns regarding their medical responsibilities, given that they could not assess the patients’ skin condition using the standard measuring instruments:

There is uncertainty, every time that there’s no physical attendance. Then it is difficult to assess how the skin might look. You might only be able to relate to what it looked like in the past, or what the patients describe. Has it gotten better or worse?

Doctor

I sort of sit for a while and think well uncertainty in relation to how their skin is, it’s also about how they feel it. Whether they are satisfied.

Nurse, FG1

As they did not have access to all the usual data, HCPs felt a loss of control and some felt a sense of insecurity. As a result, HCPs reflected on the importance of addressing patients’ perspectives on their own skin condition, treatment, and well-being. For the HCPs, it was important that the video consultations made sense to the patients and made everyday life with psoriasis easier:

I don’t want to be a nurse if I only get to sit and talk to patients over a video. It’s a bit ambivalent, because I think it’s really good for the patients. They don’t have to drive for several hours to get here.

Nurse, FG2

The patients’ needs took precedence over the HCPs’ preferences to see patients at in-person visits. For some, the video consultation could fulfill this wish to some extent:

It just provided something completely different, being able to see him. Because you don’t know, when you call them. You have no idea, if it is someone you don’t know, how they are feeling. And it just provided something, that one could talk and laugh a little and so I am also positive about it.

Nurse, FG2

Furthermore, HCPs emphasized that video consultations should be based on patients’ needs and that patients should be made to feel safe and confident.

Although the HCPs had some concerns regarding their medical responsibilities, patients felt a sense of confidence related to there being no need to attend quarterly follow-ups:

I’m following a course of treatment and I can of course see on my own body. I don’t need to see what the numbers and all that say.

IV, P6

As patients had lived with a chronic disease for many years and were now on treatment with a significantly higher effect (biological treatment), they felt confident and were capable of self-assessing their needs at either a follow-up in person or by video. In this way, patients expressed independence and were willing to take on responsibility.

Giving patients the chance to have video consultations provided them with a degree of freedom related to everyday life and work:

I experienced it like going to a meeting where you have to show up, but within parameters where you can do what suits you. I was at home so it was super easy...so I could just be by myself. I have to use a whole day more or less [for in-person visits]. So I can save 2 days and spend them on something else. That’s what’s positive about having these video consultations.

IV, P3

When patients attended video consultations, there was peace and quiet during and around the consultations. Not having to take a day off work and spend a day on traveling was regarded as an advantage, both personally and financially. Thus, using the app allowed for flexibility, which was highly appreciated by the patients. That said, patients used it when they needed to and when it was beneficial to their everyday lives. Along with the unaccustomed and appreciated attentiveness through video consultations, the mHealth solution was experienced as an improvement in the management of psoriasis.

### A New Approach Requires New Competencies

Using a new technology during follow-up consultations required that the HCPs acquire new competencies and working procedures. Regardless of whether the consultations were conducted by video or in person, they had to manage the digital responses to the questionnaires:

I have to say as well that it is still so new for me, I looked at the questionnaires and then I forgot everything about what they wrote.

Nurse, FG1

The new workflows and procedures were not a part of the existing consultation routines, and this meant that some completed questionnaires were not addressed or were not followed up. For the patients, this was somewhat annoying, although they recognized that it was a new workflow, and thus, they seemed to be prepared for difficulties during the test:

I had also written all my numerical values/data and things like that, but I don’t know if it wasn’t up and running or something, but in any case they had not checked them, but I’m sure it will come.

IIP, P2

Another difficulty during the test was that not all the HCPs seemed familiar with all of the app’s content and features. If patients were not told about the app capabilities, they did not know to register their personal data, such as blood pressure, pulse, and weight. The focus was on the questionnaires, and during a busy workday, HCPs forgot to inform patients about all of the app’s features. However, they also expressed the need to become much more familiar with the technology and its possibilities.

For the HCPs, it seemed important that the video consultations required more structure and that there should be some considerations about how many patients should be booked and at what time during the day:

Yes, if one had all telemedicine patients gathered on one day. Or one morning, or late in the day. Then you can take them one after the other. However, if they are booked in between the other patients, there is always a great risk of getting delayed. Because you don’t know, just before, what kind of patient you are getting. Then you get more pressured.

Doctor, FG1

The pressure on workflow was caused by the fact that video consultations were not part of the routine, and therefore, the HCPs did not know what to expect. In addition, because consultation by video had not yet become routine, they were not sure how best to round off consultations, which also added some pressure:

When it is a video, it takes longer, because the patients have such a desire to talk. So, maybe it has opened up more. And for some [HCPs] it has also been a bit hard to round off.

Nurse, FG2

There was overall agreement between HCPs that the app should be offered to all patients with psoriasis; however, there were reflections on which patients should be offered video consultations. HCPs were reluctant to offer video consultations to the patients categorized as complicated:

But if it is a complicated patient, then I don’t want to mention it.

Doctor, FG1

The HCPs’ individual and subjective assessments of the patient would define whether the patient was complicated. This could be related to patients with social challenges or lifestyle-related diseases. Nevertheless, if a complicated patient requested a video consultation, the HCPs said they would offer it. When they first started recruiting patients for video consultations, there was a tendency to include patients who lived far away from the hospital. With time, however, there was a growing recognition that this was not an optimal strategy:

Yes, yes, but that about not even offering it because one thinks they are over 75 and can’t work it out. I’ve had someone who himself requested it. He was 86.

Nurse, FG1

The quoted text shows that it was impossible to predict who wanted, who could benefit from, and who could manage a video consolation. This created an awareness that all patients should be considered eligible candidates, and the opportunities should be discussed with patients to identify their thoughts and perspectives on future video consultations.

## Discussion

### Principal Findings

In this qualitative study, patients experienced that their reflection on what was important for them to discuss at consultations was improved, through preparation using questionnaires that were filled out electronically at home. The questionnaires gave them the opportunity to ask questions ahead of a consultation. In a Cochrane review, interventions that helped patients ask questions and gather information before consultations resulted in small increases in questions asked and patient satisfaction [20]. Being able to ask questions and take responsibility for your health is an important part of self-management. The consultation, and thus the relationship between patients and their HCPs, is essential and should facilitate patient participation in decision making. Including patients’ agendas in consultations is important, but the limited communication between patients and HCPs seems to challenge this [21] and needs to be strengthened [22]. Using an app that included preparation and contributed to the agenda, had a positive effect on patients’ perceptions of the collaboration between HCPs and patients. This is consistent with another study that found that patients’ reflection and collaboration with HCPs was improved by the use of an app that used questions as a preparation for consultations, as it gave patients a voice in consultations [23]. The use of questions to prepare ahead of consultations allowed patients to contribute to their individual experiences and gave them the opportunity to address aspects other than medical ones [23]. This could indicate that patients’ involvement before and during video consultations could be a way to strengthen the relationship.

This study found that video consultations provided patients with a degree of freedom to better balance everyday life with psoriasis. In relation to other chronic conditions, telehealth is perceived by patients to be convenient and leads to them feeling more involved in decisions about their care and greater confidence in managing their own health [24]. Our patient participants experienced independence and were willing to take on responsibility for not having to physically attend follow-ups every 3 months. By allowing them to decide whether they wanted to participate in video consultations, patients were encouraged to become active in their care. Supporting patients in this active role or active engagement can be seen as a step toward a more patient-centered approach [25].

Some patients and HCPs were unaccustomed to video consultations and required more adaptation, which is consistent with other findings [26]. According to postphenomenology, as described by Ihde [27], dealing with technology in the field of health care is a process. There must be room for resistance and adaptation of the technology in the interaction with humans in practice. Ihde [27] uses the term embodiment to describe the integration of a technology. It refers to the process that occurs when a given technology becomes integrated as a useful tool for those who use it. Thus, postphenomenology deals with how a technology shapes the relationship between humans, where technology is not regarded as a neutral force [28]. Technological mediation, constitution, and multistability are the concepts used to describe this relationship [28]. The mHealth solution in this study enabled both preparation ahead of consultations and video consultations that mediated a new way of interaction between patients and HCPs. Although most patients felt confident in video consultations, some HCPs were concerned about the limited access to patient data and that they could not measure and assess patients in the usual way. In this way, the technology mediated a reflection on the importance of addressing the patients’ perspectives on how they experience their skin, treatment, and well-being. Sometimes, the completed questionnaires were not addressed by HCPs during the consultation, leading to the risk of a lack of patient-centeredness. This supports the postphenomenological concept of multistability, in that the mHealth solution can have different meanings and purposes for different users in different contexts.

Patients undergoing biological treatment are closely monitored with blood tests, skin examination, and quality of life measurements. Although the DLQI questionnaire was embedded in the TD solution and access to patients’ blood samples was available, we found that the missing skin examination was a concern for HCPs. Patients’ safety is an important aspect in the field of TD, especially when a TD solution, such as the one in this study, may replace some routine consultations. However, decreasing in-person visits for patients receiving biologics has shown no harm in patient safety or monitoring, but instead, it provides patients with more flexibility [29]. In this study, we did not focus on replacing routine in-person consultations, but on facilitating a more patient-centered approach in clinical practice through the use of technology. In our study, the use of video consultations was requested by patients during phase 2 of a PD process [7].

Another finding was that HCPs offered the mHealth solution to patients they perceived as not complicated or to patients living far away from the hospital. It is often argued that telehealth reduces inequality in health by increasing access to health services [30]. Furthermore, the use of a PD, as in this study, is recommended to further reduce these inequalities [31]. However, if HCPs subjectively choose eligible candidates, this could increase social health inequalities, as it automatically excludes a certain group of patients. During the test period, some HCPs became aware that their approach to patient selection might have been wrong, as they found that certain patients, who they would not have considered including, asked for a video consultation. In this way, the HCPs were confronted with some of their existing prejudices and reflected on how to solve this problem. Nevertheless, this is a barrier for the full embodiment of the mHealth solution, and a clear structure and alignment regarding who to include is required.

### Limitations

A limitation of this study is that it was a single-center study that included 14 patients and 9 HCPs, which is a rather small sample size. However, this was an evaluation of an mHealth solution whose aim was to explore the experiences and perceptions of patients and HCPs who used it. This lends itself to a qualitative approach, and thus, the sample size seems adequate, as qualitative research is concerned with the deepening and understanding of a phenomenon, rather than with numerical representability [32]. In addition, we aimed for maximum variation during participant recruitment and included patients aged 27-67 years, which is considered a strength [33]. Telephone interviews used in qualitative research are not the most common method for data generation because of the loss of contextual data, such as nonverbal communication. However, there seems to be limited evidence that telephone interviews, with certainty, lead to data loss [14]. Furthermore, this study did not collect sensitive data, and the participants were familiar with the author conducting the interviews.

### Implications for Practice

An mHealth app for patients with psoriasis receiving biological treatment can be used at follow-up consultations. However, it should be used as a solution to support both HCPs and patients to facilitate a patient-centered approach and increase patients’ self-management. Thus, an mHealth solution has the potential to improve health management of this patient group.

### Conclusions

The mHealth solution, in the form of an app, has the potential to strengthen the relationship between HCPs and patients and for patients to become more involved in their care. The mHealth solution was considered easy to use and facilitated support and reflection among patients, as it gave patients the opportunity to prepare ahead of consultations. Video consultations provided patients with a degree of freedom to better balance their everyday life with psoriasis. However, alignment and a clear structure with regard to patient selection as eligible candidates for video consultations are required to reduce social health inequalities. In addition, video consultations changed the HCPs’ work practice, necessitating new types of skills to communicate with patients.

### Future Study

The mHealth solution has an impact on clinical practice, and to ensure its sustainability and increase its use, initiatives need to be designed to start the implementation process.

## Abbreviations

DLQI: Dermatology Quality of Life Index
HCP: health care professional
mHealth: mobile health
PD: participatory design
TD: teledermatology
